# Intergenerational response to sperm competition risk in an invasive mammal

**DOI:** 10.1098/rspb.2022.2452

**Published:** 2023-04-26

**Authors:** Renée C. Firman, Gonçalo Igreja André, Jessica H. Hadlow, Leigh W. Simmons

**Affiliations:** ^1^ Centre for Evolutionary Biology, School of Biological Sciences, University of Western Australia, 35 Stirling Highway, Crawley, Western Australia 6009, Australia; ^2^ Department of Biology, University of Maryland, College Park, MD, USA

**Keywords:** sexual selection, *Mus musculus domesticus*, pest species, fertility control, male–male competition, phenotypic plasticity

## Abstract

Studies of socially mediated phenotypic plasticity have demonstrated adaptive male responses to the ‘competitive’ environment. Despite this, whether variation in the paternal social environment also influences offspring reproductive potential in an intergenerational context has not yet been examined. Here, we studied the descendants of wild-caught house mice, a destructive pest species worldwide, to address this knowledge gap. We analysed traits that define a ‘competitive’ phenotype in the sons of males (sires) that had been exposed to either a high-male density (competitive) or high-female density (non-competitive) environment. We report disparate reproductive strategies among the sires: high-male density led to a phenotype geared for competition, while high-female density led to a phenotype that would facilitate elevated mating frequency. Moreover, we found that the competitive responses of sires persisted in the subsequent generation, with the sons of males reared under competition having elevated sperm quality. As all sons were reared under common-garden conditions, variation in their reproductive phenotypes could only have arisen via nongenetic inheritance. We discuss our results in relation to the adaptive advantage of preparing sons for sperm competition and suggest that intergenerational plasticity is a previously unconsidered aspect in invasive mammal fertility control.

## 1. Introduction

Impactful events experienced during offspring development, such as malnourishment or severe stress, have been reported to influence phenotypic expression in adulthood [1–4]. In these cases, perturbation of the parental (F0) environment has directly affected the developing (F1) generation. However, offspring phenotypes may also be mediated by environmentally induced epigenetic changes in parental germ cells prior to conception. Incredibly, the exposure of parents to salient environmental stimuli has been shown to influence offspring phenotypic expression, via non-genetic inheritance, when stimuli are detected prior to conception [5]. Information transfer of this type, referred to as intergenerational or transgenerational transmission, represents an efficient mode by which parents can ‘prepare’ their offspring for specific environmental conditions [6]. Intergenerational plasticity has been observed to manifest in different morphological, behavioural and physiological traits across diverse organisms, from tolerance to nitrogen-limited conditions in thale cress [7], to starvation resilience in a nematode [8], to fear sensitivity in mice [9]. These examples highlight the adaptive nature of intergenerational plasticity; parents essentially arm their offspring with a phenotype that promotes resilience in a similar environmental context [6,10,11]. Alternatively, intergenerational plasticity can also be non-adaptive noise caused by physiological processes affecting the epigenetic mechanisms in the parental germline as a response to specific environmental conditions (see [12] for discussion on the potential for non-adaptive paternal effects via sperm).

Survival is a key component of individual fitness, but so too is reproduction. In the context of socially induced plasticity, the developmental environment is recognized to have profound effects on the reproductive phenotype in adulthood. Such effects can influence mechanisms of sexual selection: a phenomenon that is exemplified by studies demonstrating specific mate choice preferences among females reared in different social contexts [13–15]. Moreover, exposure to different levels of perceived male–male competition during development can lead to adaptive responses in males. For example, males reared in the presence of (more) competitors, or cues that signify competitor presence, have been shown to develop a larger body size and/or produce more sperm (often estimated using relative testes size) at sexual maturity [16–26]. These results may be interpreted as being adaptive in a high-male density environment: larger body size may contribute to success in male–male combat or facilitate effective monopolization of females or mate guarding [27], and high sperm numbers may translate to success in sperm competition [28,29]. Research on male rodents has shown that exposure to adverse social experiences during development can affect brain and behaviour in the next generation (reviewed in [30]), raising the intriguing possibility that offspring reproductive phenotype may also be influenced by the paternal social environment. However, the effect of the paternal social environment on offspring reproductive potential has never been examined in an intergenerational context.

The house mouse (*Mus musculus domesticus*) has proven to be a productive model for studies of socially mediated phenotypic plasticity in the context of male–male competition [16–18,22,23,31,32]. Key to these investigations has been the use of urinary odours—the species' main medium for communication—to simulate variation in the social environment. Mouse urine contains both ‘fixed’ genetic information (sex, individual identity) and ‘variable’ information, the latter conveying an individual's current social, reproductive and health status [33]. Male-specific urinary volatiles are known to stimulate aggression between males [34] and are highly attractive to females [34]. Male house mice are highly territorial and continually deposit urinary scent marks to deter competitors from their defended area [35]. Mouse behaviours, including social interaction, learning, fear and stress, are closely associated with olfactory function [36]. Olfactory conditioning experiments have been particularly useful in addressing when and how the olfactory experience of parents influences the adult phenotype of the offspring [9,37].

Here, we used house mice to explore how the paternal social environment influences the reproductive phenotype of sons. Specifically, we analysed traits that are known to be important in male–male competition of sons born to males (sires) that had been reared in either a high-male density (competitive) or high-female density (non-competitive) environment. Consistent with previous investigations [16–18,22,23,31,32], we found that sires developed a phenotype that was suited to the prevailing social conditions; those exposed to a high-male density environment were larger and produced better quality sperm compared with sires exposed to a high-female density environment. Moreover, we found that this anticipatory response to sperm competition persisted in the next generation, with the sons of males reared under high-male density conditions also producing higher-quality sperm. As the sons themselves were reared under common-garden conditions, we can conclude that these differences were attributable to intergenerational transmission. We discuss our results in relation to the adaptive advantage of preparing sons for male–male competition and consider potential mechanisms underlying intergenerational transmission of sperm traits. We highlight the relevance of our findings in relation to a previously unconsidered aspect in fertility control of invasive rodent pests.

## 2. Material and methods

### (a) Experimental model and common-garden breeding

Wild house mice (*Mus musculus domesticus*; *n* = 22 individuals = 11 mating pairs) were captured and removed from Boullanger Island, located off the coast of Western Australia (30°18′55″ S, 115°00′13″ E), and transported to the University of Western Australia. Here, the mice were outbred for two generations under standard laboratory conditions. Common-garden maintenance and breeding conditions were applied to eliminate potential environmental factors that may affect the traits of interest in the current study. Thus, mice were housed individually in standard cages (16 × 33 × 12 cm) and maintained at a constant temperature (24°C) on a reverse light–dark cycle (14 h : 10 h). All animals were provided with food and water ad libitum and experienced the same husbandry routine. Matings were conducted during the dark phase under a red light [38]. Females were checked regularly to detect oestrus [39]. When females were in oestrus, matings were initiated with the introduction of a female into a male's cage. The female was then inspected half-hourly for the presence of a mating plug. We used the presence of a mating plug as an indicator of a complete, successful mating event [38]. After mating, each female was placed in a clean box with shredded paper for nesting and left undisturbed until parturition. The litters were weaned at three weeks of age, at which time the experimental males (= sires; third generation born in the laboratory) were exposed to different social environments as described below. An overview of the experimental design is provided in electronic supplementary material, figure S1).

### (b) Social environment manipulation (F0) and common-garden rearing (F1)

Olfaction is the primary sensory modality in house mice, and conspecifics are recognized by individually distinct scent signals [35]. It is known that aspects of social communication in male mice largely depend on the activation of receptor neurons in the vomeronasal organ (VNO) by components of urine [40]. Specifically, the VNO is required for both sex discrimination [41] and the detection of odorants that determine mate preferences [42] (i.e. genetic mutations of different regions of the VNO have resulted in aberrant sexual and social behaviour; [42]). In this investigation, we used the reliance of urinary odour for social communication to manipulate the social experience of males during their sexual development via differential exposure to rival male and female scents. That is, we exposed sires (F0) to different social environments using established protocols known to induce phenotypic plasticity in the reproductive traits of house mice [16–18,31,43,44]. We controlled the overall density of individuals that sires were exposed to while manipulating their exposure to male and female urinary odours [18,44]. As house mice can recognize conspecifics via individually distinct odour signals [35], our methods ensured that males experienced one of two different social environments during their sexual development [17,18]. Sires were reared in either a high-male density (*n* = 35) or high-female density (*n* = 35) environment (under a paired design with one brother of each litter used in each treatment to control for family-derived variation). The different social environments were created by housing sires in standard cages placed on metal racks in two separate constant temperature rooms (CTRs) (electronic supplementary material, figure S1). In the high-male density environment, sires were reared from 3 to 15 weeks within close proximity to other males, consisting of both sexually mature males and males of the same age (see electronic supplementary material, figure S1). Sires were rotated through the rack positions on a regular schedule. Twice a week, each sire was exposed to 15 g of urine-soiled chaff from 16 sexually mature males. Once a fortnight, each sire experienced a ‘male encounter’. Thus, each sire was released into a large, plastic opaque tub (49 × 74 × 41 cm) containing two sexually mature males. The males remained housed in their cages for the duration of the encounter. Thus, sires roamed freely in the tub for 30 min, but could only interact with the males through the wire cage lids. The sires were exposed to different males in each encounter. To ensure normal reproductive development, the sires were periodically exposed to the urinary odours (soiled chaff) of the 16 mature females from the non-competitive environment.

The high-female density environment was established in a second CTR. Here, sires were reared from the age of 3 weeks to 15 weeks within proximity to females, consisting of sexually mature females and females of the same age (see electronic supplementary material, figure S1). As in the high-male density environment, sires in the high-female density environment were rotated through the rack positions on a regular schedule. Twice a week, each sire was exposed to 15 g of urine-soiled chaff from 16 sexually mature females. Sires experienced fortnightly ‘female encounters’, which were conducted as described above, but with males able to interact with two sexually mature females through wire cage lids for 30 min (electronic supplementary material, figure S1). The high-male density and high-female density treatments were swapped between the two CTRs midway through the experiment to avoid potential ‘room’ effects.

The sires were exposed to the high-male density (*n* = 35) and high-female density (*n* = 35) conditions until sexual maturity (approx. 100 days of age), at which time they were mated with females to produce a subsequent generation (F1). Sires that were brothers were split across treatments and mated with females that they were unrelated to but that were from the same family (i.e. sisters) to control for family-derived variation of maternal origin. These females were from the same generation as the sires and had been reared under common-garden conditions in a separate room from the experimental rooms. Breeding was conducted under common-garden conditions as described above: (i) females were checked regularly to detect oestrus [39], (ii) matings were initiated with the introduction of a female into a male's cage, (iii) females were inspected half-hourly for the presence of a mating plug, and (iv) after mating, females were placed in a clean box with shredded paper for nesting and left undisturbed until parturition. The sires were returned to their treatment and then euthanized via cervical dislocation 9 days after mating, which allowed for the full replenishment of sperm stores [45], and anatomical measurements and sperm analyses were performed. The F1 litters were weaned at three weeks of age, at which time the F1 males were housed individually until sexual maturity. Up to four sons from each litter were kept for the experiment (*n*_sons_ = 100 from *n*_sires_ = 69; i.e. one sire died prior to mating). Sons were reared under common-garden conditions during the developmental period; they were provided with food and water ad libitum and experienced the same husbandry routine. From weaning, the F1 males were arranged in rows consisting of four individually housed sons and two pairs of females (sisters where possible), with sons always housed next to one female pair. Male cages were rotated indiscriminately each fortnight to account for differences in rack positions (rack configuration was maintained). Once the sons had reached sexual maturity (approx. 100 days of age), they were euthanized via cervical dislocation, and anatomical measurements and sperm analyses were performed.

### (c) Anatomical measurements and sperm analyses

Anogenital distance (AGD; sons only) was measured using digital callipers prior to euthanasia. Immediately following euthanasia, body mass, testes mass and seminal vesicles (SV) mass were measured using an electronic balance. The epididymis was dissected to extract sperm according to published protocols routinely performed in our laboratory [38,46]. Specifically, both the left and right caudal epididymides were incised, placed in 1 ml of human tubal fluid (HTF), and incubated at 37°C. Following an initial 10 min incubation period, which allowed the sperm to swim into the medium, the epididymal tissue was removed from the HTF and the sperm suspension was incubated for a further 50 min (37°C). Sperm quantity and quality were ascertained using the CEROS II computer-assisted sperm analysis system under standard mouse sperm parameters (v. 1.3, Hamilton Thorne Research). For this, approximately 10 µl of sperm suspension was loaded into a haemocytometer and ten fields of view were scanned. The values produced by the replicate scans were highly repeatable (electronic supplementary material, table S1). We calculated mean sperm concentration (×10^6^ ml^−1^), percentage of motile sperm, percentage of progressive sperm and average path velocity (µm s^−1^) for each individual and used these values in our analyses.

### (d) Statistical analyses

Linear mixed models (LMMs) were used to analyse differences among sires and sons in (i) body mass, (ii) AGD (sons only), (iii) testes mass and (iv) SV mass based on the social environment that sires were exposed to during sexual development. We used principal component analyses (PCAs) to summarize variation among the correlated sperm traits for sires and sons (electronic supplementary material, table S2). The individual principal components (PCs) were extracted and used in a LMM to test for differences in sperm quantity and quality based on the social environment that sires were exposed to during sexual development. Family identity was included as a random effect in all models. Sire identity was also included as a random effect in the LMMs of sons. Body mass was included as a covariate in all LMMs to control for size-derived variation. We performed an additional test of the biological effects by calculating effect sizes and their 95% confidence intervals (CIs) for the anatomical traits and PC values. All analyses were conducted in R v.3.5.1 [47] using the *lmer* function implemented within the package *lme4* (LMMs) [48], the built-in *prcomp* function (sperm trait PCAs) or the *effectsize* function implemented within the package *effectsize* (Cohen's *d* and 95% CI calculation) [49].

## 3. Results

**Figure 1.**
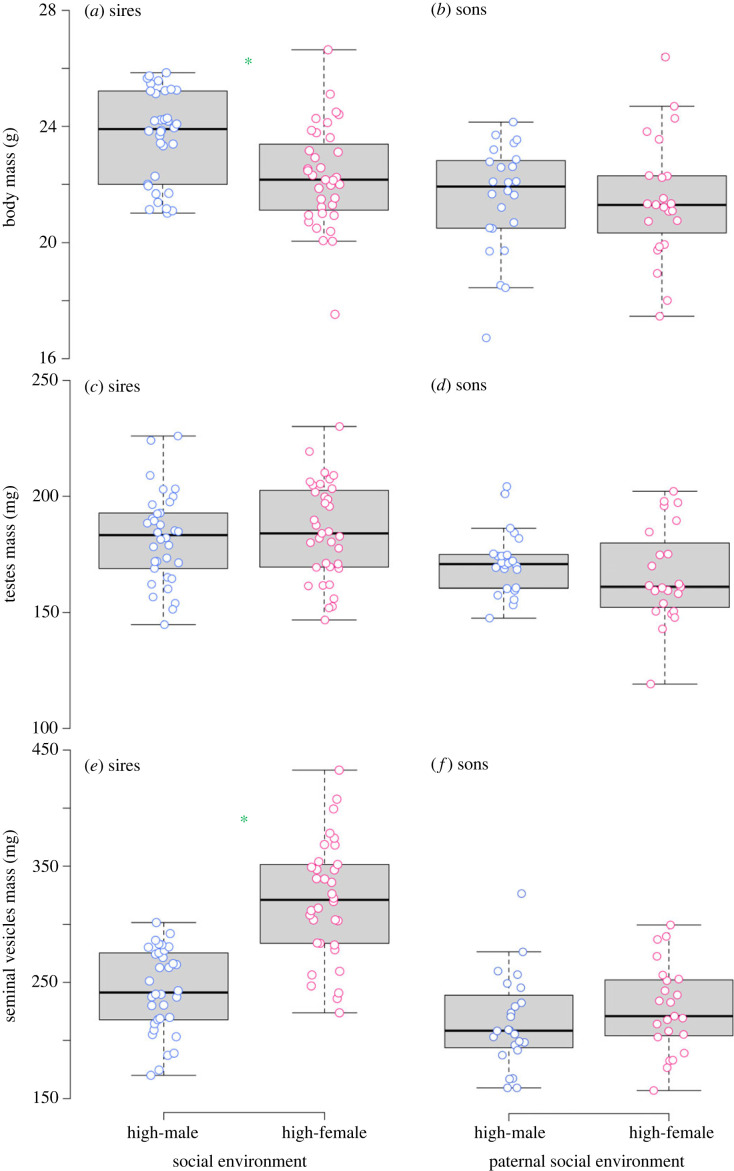
Anatomical traits of sires and sons, where sires were exposed to either a high-male or high-female density environment during sexual development. Sires that developed under high-male density conditions were larger in body size compared with sires exposed to high-female density conditions (*a*). Sires exposed to a high-female density environment developed larger seminal vesicles (SV) compared with sires exposed to a high-male density environment (after controlling for variation in body size) (*e*). Analyses performed on the sons' anatomical traits revealed that body (*b*), testes (*d*), and SV (*f*) mass did not differ according to the paternal social environment. For each trait, the median (box midline), first (lower box line) and third (upper box line) quartiles, and range excluding outliers (whiskers) are presented. Family means are displayed in the points. Significant difference (*p*-value < 0.05) is indicated by a green asterisk. Refer to main text for full details on the analyses.

**Figure 2.**
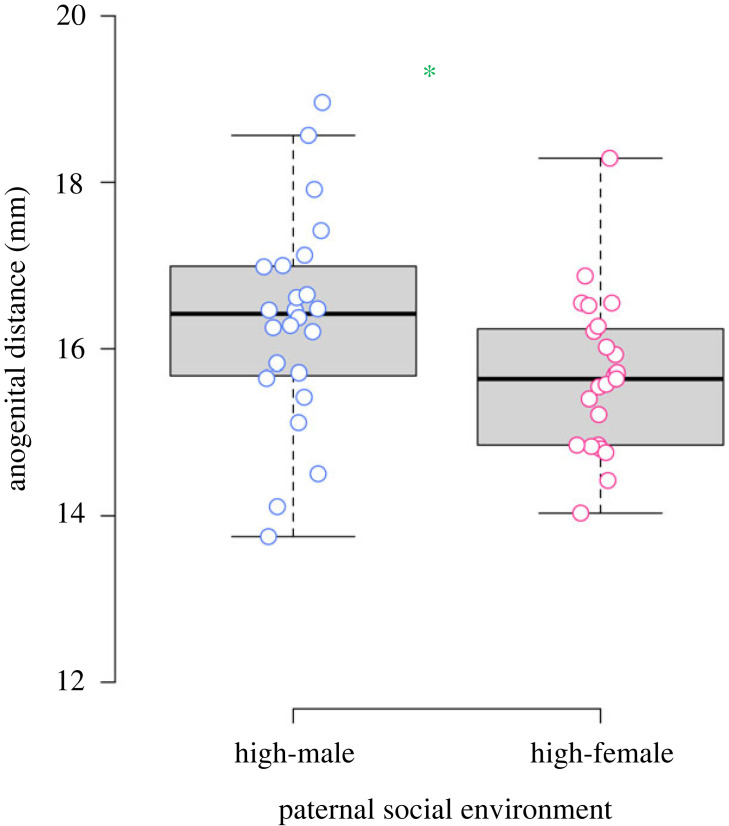
Anogenital distances (AGDs) of sons with sires exposed to either a high-male or high-female density environment during sexual development. The sons of sires that had developed under high-male density conditions had longer AGDs compared with sons of sires that had been exposed to a high-female density environment (the significant difference is indicated by a green asterisk; *p*-value < 0.05). The median (box midline), first (lower box line) and third (upper box line) quartiles, and range excluding outliers (whiskers) are presented. Family means are displayed as points. Refer to main text for full details on the analysis.

The LMMs for anatomical traits revealed that sires from the high-male density environment were larger in body size compared with sires from the high-female density environment (figure 1*a*; table 1), and that sires from the high-female density environment had larger SV compared with sires that had developed under high-male density conditions (figure 1*c*; table 1). Testes mass did not differ among sires raised in the high-male and high-female density environments (figure 1*b*; table 1). The LMMs for the sons' anatomical traits revealed that body (figure 1*b*), testes (figure 1*d*), and SV (figure 1*f*) mass did not differ according to the paternal social environment (table 2). However, the sons of males that were exposed to a high-male density environment had larger AGDs compared with the sons of males that had developed under high-female density conditions (figure 2; table 2).

**Table 1.** Linear mixed models (LMMs) investigating the effect of the social environment on anatomical and sperm traits of male (sire) house mice. Effect size (Cohen's *d*) and 95% confidence interval (CI) estimates are provided. Significant *p*-values (*p* < 0.05) are displayed in bold. SV, seminal vesicles.

fixed effect	estimate	± s.e.	type II, Wald *χ*^2^	*p*-value	Cohen's *d*	95% CI
(*a*) *body mass* (g)						
intercept	25.117	0.508				
environment	−1.433	0.298	23.169	**<0.001**	0.84	[0.34, 1.33]
(*b*) *testes mass* (mg)						
intercept	133.139	36.953				
environment	6.558	4.000	2.688	0.101		
body mass	1.771	1.452	1.488	0.223	−0.15	[−0.62, 0.32]
(*c*) *SV mass* (mg)						
intercept	−80.071	80.751				
environment	91.625	9.346	96.106	**<0.001**		
body mass	9.797	3.167	9.572	**0.002**	−1.74	[−2.30, −1.18]
(*d*) *sperm quality* (PC1)						
intercept	2.820	2.824				
environment	−0.764	0.376	4.140	**0.042**	0.46	[−0.02, 0.94]
body mass	−0.073	0.111	0.432	0.511		
(*e*) *sperm quantity/speed* (PC2)						
intercept	−0.458	2.366				
environment	−0.010	0.278	0.001	0.972		
body mass	0.020	0.093	0.049	0.826	0.04	[−0.43, 0.52]

**Table 2.** Linear mixed models (LMMs) investigating the effect of the sires’ social environment on anatomical and sperm traits of male (sons) house mice. Effect size (Cohen's *d*) and 95% confidence interval (CI) estimates are provided. Significant *p-*values (*p* < 0.05) are displayed in bold. AGD, anogenital distance; SV, seminal vesicles.

fixed effect	estimate	± s.e.	type II, Wald *χ*^2^	*p*-value	Cohen's *d*	95% CI
(*a*) *body mass* (g)						
intercept	21.581	0.863				
sire environment	−0.060	0.546	0.012	0.912	<0.01	[−0.38, 0.40]
(*b*) *AGD* (mm)						
intercept	12.026	1.440				
sire environment	−0.682	0.276	6.107	**0.013**		
body mass	0.230	0.064	13.038	**<0.001**	0.50	[0.10, 0.89]
(*c*) *testes mass* (mg)						
intercept	68.892	25.574				
sire environment	−5.335	4.514	1.397	0.237		
body mass	5.031	1.141	19.444	**<0.001**	0.20	[−0.20, 0.59]
(*d*) *SV mass* (mg)						
intercept	−49.301	52.890				
sire environment	11.147	9.469	1.386	0.239		
body mass	11.869	2.354	25.419	**<0.001**	−0.23	[−0.62, 0.17]
(*e*) *sperm quality* (PC1)						
intercept	2.700	1.635				
sire environment	−0.688	0.302	5.194	**0.023**		
body mass	−0.074	0.073	1.041	0.308	0.48	[0.09, 0.88]
(*f*) *sperm quantity/speed* (PC2)						
intercept	3.405	1.241				
sire environment	0.034	0.250	0.019	0.892		
body mass	−0.161	0.055	8.702	**0.003**	−0.09	[−0.49, 0.30]

**Figure 3.**
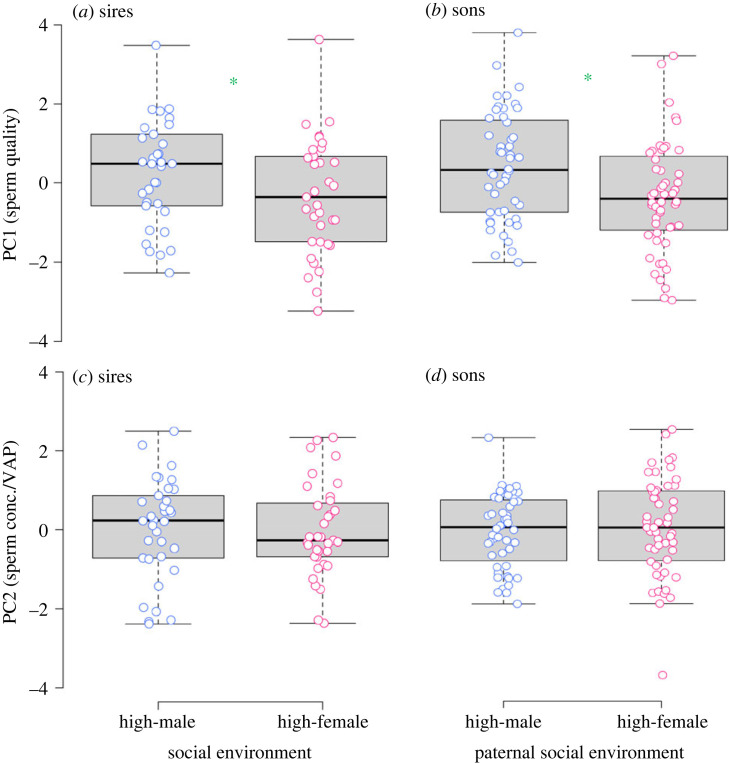
Principal component analysis (PCA) was applied to summarize variation in sperm traits of sires and sons, where sires were exposed to either a high-male or high-female density environment during sexual development. For both sires and sons, the traits contributing most to PC1 were percentage of motile and progressive sperm (*sensu* sperm quality), and the variables contributing most to PC2 were sperm concentration and average path velocity (VAP) (see main text for full details on the PCA). A linear mixed model (LMM) revealed that sires that developed under high-male density conditions had larger PC1 values compared with sires exposed to high-female density conditions (*a*). The sons of males that developed under high-male density conditions had larger PC1 values compared with the sons of sires exposed to high-female density conditions (*b*). There was no effect of treatment on the PC2 values of sires (*c*) and sons (*d*). The median (box midline), first (lower box line) and third (upper box line) quartiles, and range excluding outliers (whiskers) are presented. Individual values are displayed as points. Significant difference (*p*-value < 0.05) is indicated by a green asterisk. Refer to main text for full details on the analyses.

The sperm PCAs produced two principal components (PCs) with eigenvalues greater than 1, which cumulatively explained 92.9 and 84.7% for the sire and son analyses, respectively (electronic supplementary material, table S3). In both generations, the variables contributing positively to PC1 were percentage of motile and progressive sperm (*sensu* sperm quality; see [50], which demonstrates that increased sperm motility leads to greater competitive fertilization success) and to a lesser extent the total concentration of sperm in the sample (electronic supplementary material, table S3). The variables that contributed most to PC2 were sperm concentration and average path velocity, describing samples with a low concentration of sperm with high average path velocity (electronic supplementary material, table S3) [51]. A LMM revealed an effect of social environment on PC1, whereby sires exposed to a high-male density environment produced greater concentrations of motile and progressive sperm compared with sires exposed to a high-female density environment (table 1; figure 3*a*). This result was also reflected in the F1, with the sons of sires exposed to a high-male density environment on average having greater PC1 values, corresponding to greater concentrations of motile and progressive sperm, compared with sons of sires exposed to a high-female density environment (table 2; figure 3*b*). The LMMs revealed that social environment did not influence sperm concentration or average path velocity (PC2) in the sires (table 1; figure 3*c*) or the sons (table 2; figure 3*d*).

The calculated effect sizes were consistent with the results obtained from the LMMs. For the sires, the effect size was very large for SV mass and large for body mass (95% CIs do not contain zero), with a medium effect size for PC1 (95% CIs marginally contain zero) (table 1). The effect sizes for testes mass and PC2 were very small (95% CIs are centred around zero) (table 1). For the sons, we report medium effect sizes for AGD and PC1 (95% CIs do not contain zero), while the effect sizes for all other traits were very small to small (95% CIs are centred around zero) (table 2).

## 4. Discussion

In this investigation, we studied house mice to examine how specific features of the paternal sensory environment before conception influence the reproductive phenotype in the subsequently conceived F1 generation. Parental responses may be anticipatory in the sense that phenotypic change is initiated before the appearance of an environmental factor or social situation by assessing stimuli or cues that are predictive of future conditions [52]. In line with this, and consistent with our previous research [16–18,31,32] as well as the work of others [22,23], we found that males (sires) exposed to the odours of many competitors developed a ‘competitive’ phenotype, consisting of larger body size and producing better-quality sperm, compared with males that had developed in a non-competitive, high-female density environment. Interestingly, testes size did not differ among males reared in environments with variation in the perceived level of male–male competition. This result is consistent with our previous investigations [16,17,31,32], differing only from one [18]. It may be that the rate of sperm production, for example via the proportions by which the testes are ‘packaged’ with sperm-producing and non-sperm producing tissue [16], is increased without increases in testes size [22,53,54]. In house mice, increased body size is associated with enhanced fighting ability and dominance status, and therefore contributes to success in premating competition [55], whereas high proportions of motile sperm are critical to competitive fertilization success [50]. Be it energy spent on defending a territory against a rival or the requirement to produce better-quality sperm to gain a fertilization advantage over a rival, competition is inherently costly to males [56,57]. Plasticity in body size and sperm quality therefore enables males to invest strategically in these traits: invest more when competition is expected to be frequent or intense and less when the risk of competition is low. It is interesting that sires in the non-competitive, high-female density environment developed larger SV compared with sires from the competitive, high-male density environment. SV contribute the major portion of the seminal fluid in which mammalian sperm are bathed during ejaculation [58]. Specifically, seminal vesicle fluid plays an integral role in the formation of the copulatory plug and provides nutritive and physiochemical support to sperm during transit within the female reproductive tract [58]. Our results may therefore represent disparate strategies corresponding to variation in the social environment; high-male density leads to a phenotype geared for competition, while high-female density leads to a phenotype that facilitates elevated mating frequency. This finding also highlights the intriguing potential for the requirement of males to trade-off between seminal fluid and sperm components of the ejaculate [59].

There is increasing evidence to show that the paternal environment can have intergenerational effects on offspring [60,61], which may be adaptive [62,63] or non-adaptive [64,65]. Specifically in mice, paternal fear conditioning leads to the same fear response in subsequently conceived sons [9] and paternal diet has been shown to have marked effects on the metabolic physiology of sons conceived after the sire's diet has been manipulated [66]. In both cases, the transmission pathway was linked to sperm epigenetic regulation [9,66]. Here, we discovered that the paternal social environment has lasting, intergenerational implications for sons. We found that the sons of males reared under high-male density conditions produced high-quality ejaculates, containing elevated proportions of progressively motile sperm, compared with the sons of males reared under high-female density conditions. In a previous investigation that adopted the same experimental design applied here, we found that male house mice that developed under high-male density conditions had longer AGDs than males that developed in a high-female density environment [18]. In the current study we did not measure sire AGD; however, we did take these measurements on the F1 males and found that the sons born to sires reared under high-male density conditions had longer AGDs compared with sons born to sires reared under high-female density conditions. Longer AGDs are linked to elevated testosterone levels, and testosterone level mediates scent-marking behaviour [67]. Thus, it seems that a high-male density environment induces high testosterone levels (longer AGDs), which may indicate more frequent scent marking behaviour, in both sires and sons. All sons were treated identically during sexual development by being reared under common-garden conditions. We can therefore be confident that the sons' reproductive phenotype was inherited, and not socially transmitted, from the F0 generation. If sons were exposed to an environment with a high density of male competitors, the transmission of environmental information would extend the adaptive capacity of plastic responses to different social conditions experienced by the sire. If not, the transmission of high sperm quality across generations, the production of which presumably comes at a cost to males, may be non-adaptive. The hypothesis that needs testing is whether the potential utility, and possible stability, of environmentally induced trait transmission is dependent on the offspring's prevailing environmental context.

How variation in the paternal social environment comes to be linked to F1 sperm performance is an intriguing question upon which we can only speculate. Research on epigenetic marks in sperm as possible intergenerational transmission routes has been conducted in rodents (reviewed in [30]). Among the epigenetic mechanisms that have been implicated in paternal transmission of stress effects via sperm are DNA methylation, oxidative damage to sperm DNA, histone modifications, and changes in small noncoding RNA (sncRNA) (reviewed in [30]). For example, odorants used in parental fear conditioning in mice are known to enter the circulatory system and activate odour receptors that are expressed on sperm, thus leading to similar fear responses in the F1 when presented with the same stimuli that the F0 was conditioned with [9]. In the fear study, the investigators observed that the sperm of the F0 and F1 generations both carried epigenetic marks (CpG hypomethylation in the *Olfr151* gene) that were believed to be the basis of intergenerational inheritance [9]. It is interesting to note that olfactory receptors expressed in the sperm of several mammalian species have been identified to display a pattern of expression that is consistent with a potential role in sperm physiology [68]. More specifically, a hypothesis has been developed in relation to the pattern of expression of olfactory receptors in sperm cells being involved in sperm motility control [69]. This putative mechanism may account for the inheritance of sperm motility that we observed across generations in the current study; pertinent information related to paternal conditioning to the competitive environment, which involved olfactory stimulation to abundant male odours, may be transferred to the offspring via epigenetic sperm marking. Future studies could be well served by examining whether this is the case, with the potential to use irradiation-based approaches that would be expected to nullify F0 ‘information storage’ during spermatogenesis [9].

More broadly, our results hold implications for a previously unconsidered aspect of invasive rodent fertility. House mice specifically engender major economic impacts in both developed and developing counties and in urban and rural habitats, the latter especially during population outbreaks [70]. The basis for the occurrence of population outbreaks to plague proportions has been attributed to the ability of mice to produce large litters in a short time in response to resource pulses [71]. However, social processes operating within populations of eruptive species vary considerably during different phases of their population dynamics [71]. For example, a correlation between amicable social behaviour, which improves reproductive success via communal breeding, and population growth rates has been described for female house mice [72,73]. Here, we identified that sperm quality, a trait linked to male fertility potential, can be transferred across generations via social processes. Our discovery suggests that an acute elevation in exposure to rival males during population outbreaks may contribute to increased male fertility potential across generations: potential that would perpetuate even after the intensity of the competitive environment has dissipated. Fertility control is currently the favoured management tool for managing feral mouse populations [70], and it has been suggested that maintaining infertile individuals in the population may be beneficial as they will remain to compete with fertile individuals for space, resources and social status [74]. However, our results suggest that the maintenance of infertile individuals within populations, and a corresponding elevation in male density, may lead to elevated reproductive potential of fertile males. While this idea remains to be tested, our findings indicate that current understanding of socially mediated mouse population growth is far from complete.

## 5. Conclusion

We have explored an underappreciated influence on the adult reproductive phenotype: ancestral social experience before conception. From a translational perspective, our results allow us to appreciate how the paternal experience markedly influences the reproductive phenotype of the subsequent generation. We interpret these results as highlighting how generations can inherit information about the salience of specific stimuli in ancestral environments so that their phenotype may be altered to allow appropriate environment-specific responses. While the complexities in elucidating the mechanism(s) that underlie intergenerational transmission of sperm traits extend beyond the scope of the current investigation, epigenetic modifications of DNA and sperm sncRNAs have been identified to be likely vectors at the interface between genes and environment and therefore represent an appropriate target for future research. Such endeavours may assist in improving our understanding of how social dynamics influence fertility parameters in outbreaking mouse populations, and ultimately assist in efforts to control these events.

## Data Availability

The data and code files are provided in the online electronic supplementary material [75].
